# Data on fluoride concentration levels in cold and warm season in City area of Sistan and Baluchistan Province, Iran

**DOI:** 10.1016/j.dib.2018.03.060

**Published:** 2018-03-16

**Authors:** Akazem Neisi, Majid Mirzabeygi (Radfard), Ghader Zeyduni, Asghar Hamzezadeh, Davoud Jalili, Abbas Abbasnia, Mahmood Yousefi, Rouhollah Khodadadi

**Affiliations:** a Department of Environmental Health Engineering, School of Health, Ahvaz Jundishapur University of Medical Sciences, Ahvaz, Iran; b MSc Graduate of Environmental Health Engineering, Tehran University of Medical Sciences, Tehran, Iran; c MSc Graduate of Environmental Health Engineering, Ardabil University of Medical Sciences, Ardabil, Iran; d Department of Environmental Health Engineering, School of Health, Ahvaz Jundishapur University of Medical Sciences, Ahvaz, Iran; e Department of Environmental Health Engineering, School of Public Health, Tehran University of Medical Sciences, Tehran, Iran; f Department of Environmental Health Engineering, School of Health, Ahvaz Jundishapur University of Medical Sciences, Ahvaz, Iran

**Keywords:** Drinking water, Fluoride, Sistan and Baluchestan, Iran

## Abstract

The need for fluoride in drinking water to the extent that reduces the amount of tooth decay and the other hand does not cause dental fluorosis, has been well documented as an important fact. The aim of this research is to survey values of fluoride in drinking water in Sistan and Baluchestan. In this descriptive and analytical study, the number of 551 samples during 4 seasons of 2013 year from rural drinking water sources via rural water and Wastewater Company has been taken. The concentration of fluoride in water samples was measured using SPADNS method. Results shows that the average concentration of fluoride in drinking water supplies for the rural region of Khash, Sarbaz, Iranshahr, Saravan, Nickshahr city are 0.72 (±0.31), 0.55(±0.21), 0.33 (±0.127), 0.6 (±0.24), 0.435 (±0.23) respectively.

Specifications Table

**Table:** 

Subject area	Water chemistry
More specific subject area	Water fluoride
Type of data	Tables, Figures
How data was acquired	Fluoride concentration was estimated using HACH device (spectrophotometer DR/5000 Company, USA) -Spectrophotometer (DR 5000- HACH). By SPADNS Method at wavelength of 580 nm.
Data format	Raw, Analyzed
Experimental factors	Samples were taken via polyethylene packaging with volume of 1 l from any source of drinking water provided for residents. Also samples were transported to the water laboratory in each city as soon as possible in a specified conditions
Experimental features	Determine the concentration levels of fluoride
Data source location	Sistan and Baluchestan province. Iran
Data accessibility	Data are included in this article

Value of the data

• Based on the data, Fluoridation of drinking water in rural areas with less than the WHO optimum value is recommended.
• Based on the data, in areas with low level of fluoride in drinking water, consuming food and beverages with high level of fluoride is suggested.
• Combining the reported data on fluoride concentrations in drinking water with information on ambient temperature is very useful.
• The data shown here will be informative for health policy makers by assigning interception actions against adverse health effects of fluoride with considering fluoride intake by drinking water and food.

## 1 Data

**Fig. 1:**
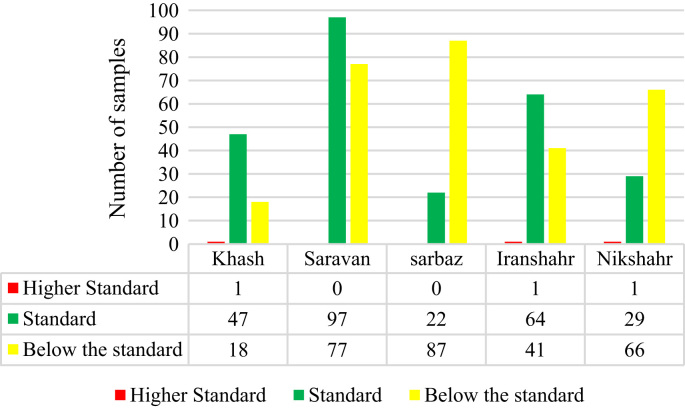
Comparison of selected parameter (Fluoride) in groundwater along with WHO permissible limits.

**Fig. 2:**
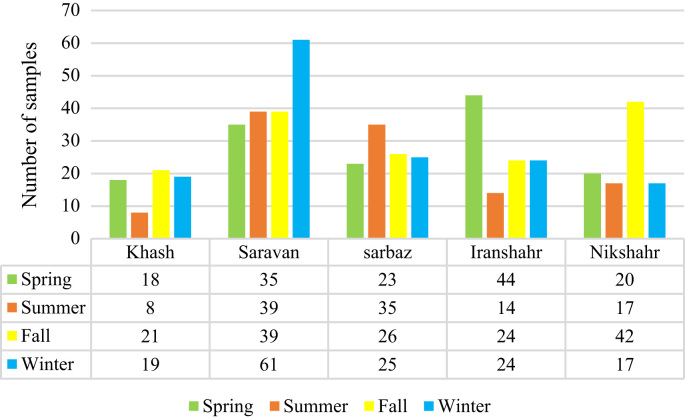
Number of samples per season in Sistan and Baluchistan province.

**Fig. 3:**
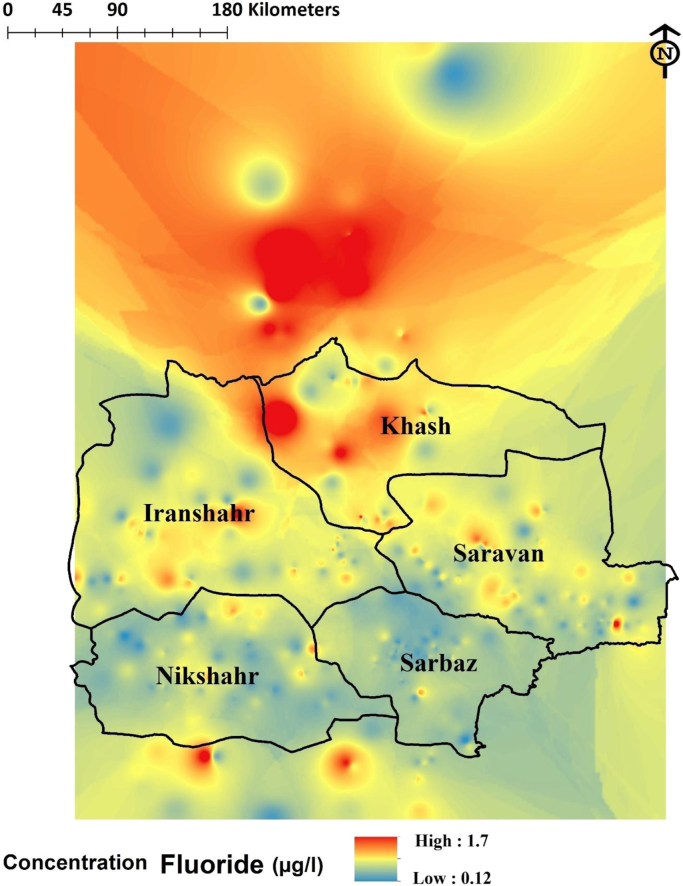
Distribution spatial Concentration Fluoride in the study area.

See Fig. 1, Fig. 2, Fig. 3 and Table 1 here.

**Table 1:** Concentrations of Fluoride and Temperature (°C) in drinking water collected from sistan and baluchestan.

Counties	Season	Mean	Min	Max	S.D.	Mean T(°C)	Min T(°C)	Max T(°C)	S.D.
Khash N = 66	Spring	0.73	0.28	1.46	0.32	30.1	23	38	3.05
	Summer	0.77	0.38	1.11	0.23	29.3	24	35	3.27
	Fall	0.58	0.39	1.71	0.2	17.3	13	38	3.4
	Winter	0.86	0.38	1.51	0.33	8.9	7	12	1.36
Saravan N = 174	Spring	0.5	0.19	0.87	0.187	38.1	32	42	2.35
	Summer	0.59	0.24	1.39	0.243	30.9	20	39	5.69
	Fall	0.5	0.01	1	0.18	19.4	12	30	4.7
	Winter	0.56	0.15	1.33	0.21	10.8	7	15	2.6
Sarbaz N = 109	Spring	0.39	0.19	0.75	0.143	31	24	36	4.05
	Summer	0.32	0.02	0.71	0.149	32.4	24.6	47	5.3
	Fall	0.3	0.01	0.5	0.18	27.5	25	35	3.45
	Winter	0.32	0.21	0.53	0.07	19.1	12	25	4.9
Iranshahr N^a^ = 106	Spring	0.57	0.21	1.26	0.02	38	18	36	4.55
	Summer	0.79	0.44	1.7	0.36	36.2	29	45	4.4
	Fall	0.51	0.23	0.87	0.165	24.7	20	32	2.78
	Winter	0.61	0.35	1.15	0.19	15.6	12	21	2.01
Nikshahr N = 96	Spring	0.4	0.14	0.67	0.15	31.6	24	39	5.1
	Summer	0.48	0.22	0.76	0.16	33	28	38	4.25
	Fall	0.44	0.19	1.55	0.29	26.5	20	39	4.1
	Winter	0.41	0.14	0.94	0.16	18	12	25	3.1

## 2 Experimental design, materials and methods

### 2.1 Study area description

**Fig. 4:**
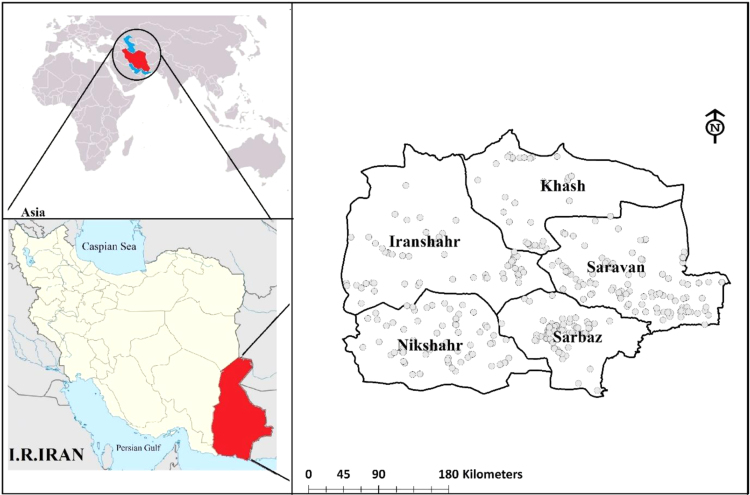
Location of water sampling sites in Sistan Baluchistan.

Sistan-and-Baluchistan province in South-East of Iran between the latitudes 25° 4′- 31°25′ N and Longitudes 58° 55′- 63°20′ E, encompassing an area is about 18175 km^2^ (Fig. 4).The area has a hot climate, and the highest and lowest air temperatures respectively are 50 °C and −7 °C, with an annual average of 25 °C.

### 2.2 Sample collection and analytical procedures

This cross sectional study has been conducted on rural drinking water sources under supervision of department of rural water and waste water province for a year. According to standard method number 2348 Institute of Standards & Industrial Research of Iran, Sampling was carried out randomly in different seasons. The number of sampling, was variable. Samples were taken via polyethylene packaging with volume of 1 l from any source of drinking water provided for residents. Also samples were transported to the water laboratory in each city as soon as possible in a specified condition. Fluoride concentration was estimated using HACH device (spectrophotometer DR/5000 Company, USA) -Spectrophotometer (DR 5000- HACH). By SPADNS Method at wavelength of 580 nm [1], [2], [3], [4], [5], [6]. Ultimately, with employing Arc GIS 9.3 software, fluoride ion dispersion at the provincial level, with using geographic coordinates (longitude and latitude regions) from collected water place, has been depicted, then description of critical points was discussed. Excel software has been used for statistical analysis of results and its comparison with national standard number 1053 Institute of Standards and Industrial Research of Iran with announcement of maximum allowable concentration of fluorides in drinking water (0.5 to 1.5 mg/l) [7], [8], [9], [10], [11].
