# Supplementary material for: Data on fluoride concentration levels in cold and warm season in City area of Sistan and Baluchistan Province, Iran
Source: Data Brief. 2018 Mar 16;18:713–8. doi: 10.1016/j.dib.2018.03.060 (PMC5996295; doi:10.1016/j.dib.2018.03.060)
Supplement: Supplementary file 1 — Supplementary material [file mmc1.docx]

The authors declare no conflict of interest.
